# Mathematical Modelling and Analysis of the Tumor Treatment Regimens with Pulsed Immunotherapy and Chemotherapy

**DOI:** 10.1155/2016/6260474

**Published:** 2016-02-22

**Authors:** Liuyong Pang, Lin Shen, Zhong Zhao

**Affiliations:** Department of Mathematics, Huanghuai University, Zhumadian 463000, China

## Abstract

To begin with, in this paper, single immunotherapy, single chemotherapy, and mixed treatment are discussed, and sufficient conditions under which tumor cells will be eliminated ultimately are obtained. We analyze the impacts of the least effective concentration and the half-life of the drug on therapeutic results and then find that increasing the least effective concentration or extending the half-life of the drug can achieve better therapeutic effects. In addition, since most types of tumors are resistant to common chemotherapy drugs, we consider the impact of drug resistance on therapeutic results and propose a new mathematical model to explain the cause of the chemotherapeutic failure using single drug. Based on this, in the end, we explore the therapeutic effects of two-drug combination chemotherapy, as well as mixed immunotherapy with combination chemotherapy. Numerical simulations indicate that combination chemotherapy is very effective in controlling tumor growth. In comparison, mixed immunotherapy with combination chemotherapy can achieve a better treatment effect.

## 1. Introduction

Immunotherapies are becoming a crucial component in the multipronged approaches which are developed to treat certain cancer [1]. By strengthening the antitumor function of the immune system, immunotherapy can enhance the body's own natural ability to combat cancer. In past decades, advances in cancer immunology have been increasingly translated into clinical testing of immune-based approaches to cancer treatment, including monoclonal antibody treatment and adoptive transfer of the cytotoxic T lymphocytes (ACT) [2, 3]. In detail, this technique [4] need identify autologous or allogeneic lymphocytes with antitumor activity, which are then infused into cancer patients, often along with appropriate growth factors (such as IL-2) to stimulate their survival. It is indispensable to identify only a small number of antitumor cells with the appropriate properties which can then be expanded to large numbers ex vivo for treatment [5]. In vitro tests can identify the exact populations and effector functions required for cancer regression, which can then be selected for expansion [6]. The cells that are activated in the laboratory can wipe off endogenous inhibitory factors and thus can be induced to exhibit the required antitumor effector functions.

Recently, clinical data have indicated that there is a potential benefit in making use of the power of the immune system in conjunction with traditional chemotherapy [7]. As a conventional treatment, chemotherapy has become a part of treatment plan of most cancer patients. Chemotherapy aims at shrinking primary tumors, slowing the tumor growth, and killing cancer cells that may have spread (metastasized) to other parts of the body from the original, primary tumor. Currently, more than 50 kinds of chemotherapy drugs are available to treat cancer and many more are being tested for their ability to destroy cancer cells [8]. Although chemotherapy is one of the principal modes of treatment for cancer patients, one of the limitations of chemotherapy is that it also kills the normal fast dividing cells, which causes serious side effects in patients.

The immune response to a tumor is usually cell mediated with cytotoxic T lymphocytes (CTL) cells and natural killer (NK) cells playing a dominant role. Mathematical modelling has become an important and useful tool in studying the interactions between the immune system and a growing tumor (Bianca et al. [9, 10], Pappalardo et al. [11–13]). Bell (1973) [14] proposed a model consisting of a system of two equations based on the classic predator-prey interaction. Kuznetsov et al., in 1992 [15] and 1994 [16], presented a mathematical model of CTL cells response to the growth of immunogenic tumor, which exhibits a number of phenomena that are observed in vivo, including immunostimulation, “sneaking through,” and “dormant state” of the tumor. Moreover, the parameters of the target model were estimated by using the experimental data of chimeric mice. de Pillis, in [1, 17–19], analyzed the interactions among tumor cells and various immune effector cells and applied the numerical calculations to discuss the treatment effects of different therapeutic regimens. Due to considering the treatment processes that are subject to short-term perturbations, the model with impulsive treatments conforms better to the practice than the continuous models mentioned above. Hence, Borges et al. (2014) [20] introduced continuous and pulsed chemotherapy to investigate the treatment effect of tumor with the help of numerical calculations.

In this paper, our purpose is to provide a useful reference and guidance for experimental workers and scientists of human cancer research by designing treatment protocols of chimeric mice with pulsed chemotherapy and immunotherapy. Hence, we introduce pulsed immunotherapy and chemotherapy into the mathematical model proposed by Kuznetsov, and the model reads

(1) dxdt=s+ρxyα+y−c1xy−d1x−α11−e−zx,t≠nτ,dydt=ry1−by−c2xy−α21−e−zy,t≠nτ,dzdt=−d2z,t≠nτ,xt+=xt+μ1,t=nτ,yt+=yt,t=nτ,zt+=zt+μ2,t=nτ,

where *x* denotes the concentration of CTL cells with antitumor activity in the tumor site, *y* represents the number of tumor cells, and *z* is the blood drug concentration. *τ* is the therapeutic period, *μ*_1_ is the infusion dose of CTL cells with antitumor activity every time, and *μ*_2_ denotes an increment of the blood drug concentration due to delivering drug at time *t* = *nτ*. *x*(*t* ^+^), *y*(*t* ^+^), and *z*(*t* ^+^) denote the right limits of *x*(*t*), *y*(*t*), and *z*(*t*) at time *t*, respectively. The descriptions and estimated values of all remaining parameters (which were estimated by using the experimental data of chimeric mice [16]) are listed in Table 1. For convenience, we suppose that immunotherapy and chemotherapy are executed at almost the same time and use TR_*i*_ = (*μ*_1*i*_, *μ*_2*i*_, *τ*_*i*_) to denote the *i*th therapeutic regimen, where *μ*_1*i*_, *μ*_2*i*_, and *τ*_*i*_ represent the dosage of immunotherapy, an increment of the blood drug concentration caused by chemotherapy and therapeutic period, respectively.

Although drug targeted therapy is yielding promising results in the treatment of some specific cancers, drug resistance caused mainly by mutation plays a critical role of the chemotherapy failure [21]. Hence, incorporating drug resistance into our model can help us to find ways to eliminate the problem. The paper is organized in the following manner. In Section 2, we will investigate therapeutic effects of immunotherapy, chemotherapy, and mixed treatment and design the corresponding therapeutical schedules. In Section 3, the efficacy of cancer chemotherapy often becomes severely limited as cancer cells become resistant to chemotherapy drugs. Hence, we will introduce the drug resistance into system (1) to account for the failure of chemotherapy and develop some new therapeutic regimens so as to achieve the goal of clinical cure. This paper ends with a brief conclusion and discussion in Section 4.

## 2. Investigation of Therapeutic Regimens

In this section, we discuss the effects of single immunotherapy, single chemotherapy, and mixed immunotherapy with chemotherapy and provide the corresponding therapeutic regimens. First of all, we discuss the single immunotherapy.

### 2.1. Single Immunotherapy

Suppose that system (1) only involves immunotherapy (i.e., *z*(0) = 0 and *μ*_2_ = 0), which is equivalent to the following system:

(2) dxdt=s+ρxyα+y−c1xy−d1x,t≠nτ,dydt=ry1−by−c2xy,t≠nτ,xt+=xt+μ1,t=nτ,yt+=yt,t=nτ.

Firstly, we give some basic properties about the following subsystem of (2):

(3) dxdt=s−d1x,t≠nτ,xt+=xt+μ1,t=nτ.

Clearly,

(4) x∗t=sd1+x∗0+−sd1exp⁡−d1t−nτ,t∈nτ,n+1τ

is a positive periodic solution of system (3), where

(5) $$\begin{array}{c} x^{*}\left(\;0^{+}\;\right)=\frac{s}{d_{1}}+\frac{\mu_{1}}{1-\exp\left(-d_{1}\tau \right)}. \end{array}$$

Since the solution of system (3) with any initial value *x*(0^+^) is

(6) $$\begin{array}{c} x\left(\;t\;\right)=\left[\;x^{*}\left(\;0^{+}\;\right)-x\left(\;0^{+}\;\right)\;\right]\exp\left(\;-d_{1}t\;\right)+x^{*}\left(\;t\;\right), \end{array}$$

we have the following lemma.

**Lemma 1 .**

System (3) has a positive periodic solution *x* ^*∗*^(*t*). And, for every solution *x*(*t*) of (3), it follows that lim_*t*→*∞*_ *x*(*t*) = *x* ^*∗*^(*t*). Furtherly, system (2) has a tumor-free periodic solution (*x* ^*∗*^(*t*), 0).

Next, we discuss the local stability of the tumor-free periodic solution (*x* ^*∗*^(*t*), 0).

**Theorem 2 .**

Let (*x*(*t*), *y*(*t*)) be any solution of system (2); then (*x* ^*∗*^(*t*), 0) is locally asymptotically stable provided that

(7) $$\begin{array}{c} \mu_{1}>\frac{\left(rd_{1}-c_{2}s\right)\tau }{c_{2}}≜\mu_{1c}. \end{array}$$

**Proof**

The local stability of the periodic solution (*x* ^*∗*^(*t*), 0) can be determined by the small amplitude perturbations of the solution. Define

(8) xt=x∗t+v1t,yt=v2t,

where *v*_*i*_(*t*)  (*i* = 1,2) are small perturbations. The linearized equations of system (2) are given by

(9) dv1tdt=−d1v1t+ρα−c1x∗tv2t,t≠nτ,dv2tdt=r−c2x∗tv2t,t≠nτ,v1t=v1t,t=nτ,v2t=v2t,t=nτ.

Let Φ(*t*) be fundamental matrix of (9) and *E*_2_ is the identity matrix; then Φ(*t*) must satisfy

(10) $$\begin{array}{c} \frac{d\Phi \left(t\right)}{dt}=\left[\begin{array}{cc} -d_{1} & \left(\frac{\rho }{\alpha }-c_{1}\right)x^{*}\left(t\right)\\ 0 & r-c_{2}x^{*}\left(t\right) \end{array}\right]\Phi \left(\;t\;\right)≜A\Phi \left(\;t\;\right), \end{array}$$

and Φ(0) = *E*_2_. Hence, the fundamental solution matrix is

(11) $$\begin{array}{c} \Phi \left(\;\tau \;\right)=\left[\begin{array}{cc} \exp\left(-d_{1}\tau \right) & \Delta \\ 0 & \exp\left(G\right) \end{array}\right], \end{array}$$

where

(12) $$\begin{array}{c} G=\overset{\tau }{\underset{0}{\int }}\left(\;r-c_{2}x^{*}\left(\;t\;\right)\;\right)dt=\frac{\left(rd_{1}-c_{2}s\right)\tau -c_{2}\mu_{1}}{d_{1}}, \end{array}$$

and the exact form of Δ is not required in this analysis.

When *t* = *nτ*, the linearization of the resetting impulsive conditions of (2) becomes

(13) $$\begin{array}{c} \left[\begin{array}{c} v_{1}\left(n\tau^{+}\right)\\ v_{2}\left(n\tau^{+}\right) \end{array}\right]=\left[\begin{array}{cc} 1 & 0\\ 0 & 1 \end{array}\right]\left[\begin{array}{c} v_{1}\left(n\tau \right)\\ v_{2}\left(n\tau \right) \end{array}\right]. \end{array}$$

Thus, the monodromy matrix of (2) is

(14) $$\begin{array}{c} Q=\left[\begin{array}{cc} 1 & 0\\ 0 & 1 \end{array}\right]\Phi \left(\;\tau \;\right)=\left[\begin{array}{cc} \exp\left(-d_{1}\tau \right) & \Delta \\ 0 & \exp\left(G\right) \end{array}\right]. \end{array}$$

Let *λ*_1_ and *λ*_2_ be eigenvalues of matrix *Q*, and then

(15) λ1=exp⁡−d1τ<1,λ2=exp⁡G.

Therefore, all eigenvalues of *Q*, namely, *λ*_1_ and *λ*_2_, have absolute values less than one if and only if (7) holds. According to Floquet theory of impulsive differential equations, the tumor-free periodic solution (*x* ^*∗*^(*t*), 0) is locally asymptotically stable.

**Remark 3 .**

The tumor-free periodic solution is unstable if *μ*_1_ < *μ*_1*c*_.

**Theorem 4 .**

A supercritical bifurcation occurs at *μ*_1_ = *μ*_1*c*_ in the sense that there is *ɛ* > 0 such that for all 0 < *ε*_1_ < *ɛ* there is a stable positive nontrivial periodic solution of (2) with period *τ* + *ε*_1_.

Detailed proof is similar to Theorem  1 in [22].

**Figure 1:**
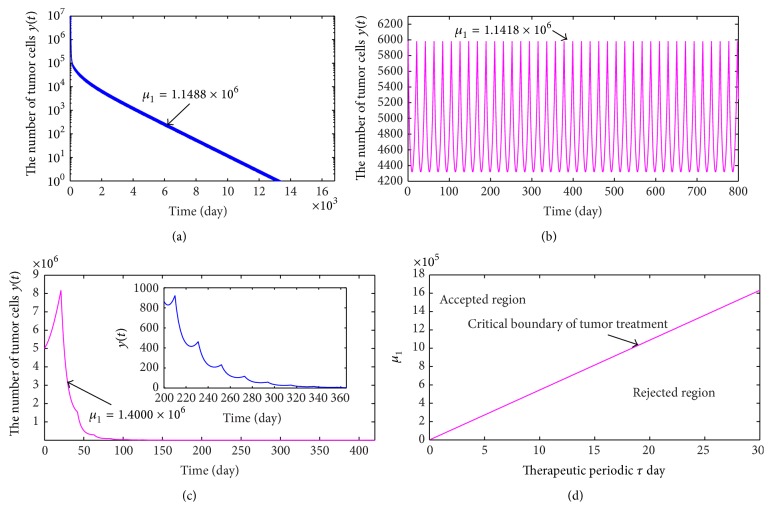
(a) shows the variation of the number of tumor cells with time when *μ*_1_ = 1.1488 × 10^6^. (b) presents the change of the number of tumor cells with time when *μ*_1_ = 1.1418 × 10^6^. (c) exhibits the changes of the number of tumor cells with time when *μ*_1_ = 1.4000 × 10^6^. The curve of dosage versus period is plotted in (d).

From (7), we know that when the infusion dose of CTL cells with antitumor activity every time is not less than the threshold value *μ*_1*c*_, then the tumor will be eliminated eventually; otherwise the number of tumor cells will present periodic oscillation. Hence, we consider a tumor with size 5.00 × 10^6^ and take therapeutic period *τ* = 21 days. From (7) and Table 1, we calculate and achieve a critical value *μ*_1*c*_ = 1.1468 × 10^6^ CTL cells. Here, we take *μ*_1_ = 1.1488 × 10^6^ (more than *μ*_1*c*_) and *μ*_1_ = 1.1418 × 10^6^ (less than *μ*_1*c*_), respectively. The variations of the number of tumor cells with time are depicted in Figures 1(a) and 1(b).  Figure 1(b) indicates that since 1.1418 × 10^6^ < *μ*_1*c*_, the number of tumor cells exhibits periodic oscillation. However, from Figure 1(a), we can see that although the tumor cells are wiped out eventually, this process takes too long time of about 37 years (13000 days). In order to shorten the time of curing a tumor, we increase the infusion dosage of CTL cells. Supposing the time of curing a cancer is a year, by numerical calculations, we achieve a new threshold value *μ*_1*c*1_ = 1.4000 × 10^6^ CTL cells. Hence, we obtain a single immunotherapy regimen:

(16) $$\begin{array}{c} TR_{I1}=\left(\;1.4000\times 10^{6}\;\;CTL\;cells,0,21\;\;days\;\right). \end{array}$$

The corresponding change of the number of tumor cells is shown in Figure 1(c) when TR_*I*1_ is carried out.

Furthermore, by Theorem 2, we derive a condition which is given by

(17) $$\begin{array}{c} \mu_{1}>\frac{\left(rd_{1}-c_{2}s\right)\tau }{c_{2}}≜L\left(\;\tau \;\right), \end{array}$$

where straight line *L*(*τ*) is defined as “critical boundary of treatment regimens.” The region which lies above *L*(*τ*) is named as “acceptable region” (i.e., the region of successful treatment), and the region which sits below *L*(*τ*) is intituled as “rejected region” (i.e., the region of failed treatment), which is shown in Figure 1(d).

### 2.2. Single Chemotherapy

We consider the case of single chemotherapy (i.e., *μ*_1_ = 0 for system (1)). Since the third equation in system (1) is independent of the variables *x* and *y*, we consider the following subsystem:

(18) dzdt=−d2z,t≠nτ,zt+=zt+μ2,t=nτ.

Clearly, system (18) has a positive periodic solution:

(19) z∗t=μ2exp⁡−d2t−nτ1−exp⁡−d2τ,t∈nτ,n+1τ,z∗0+=μ21−exp⁡−d2τ.

Since the solution of (18) with initial value *z*(0^+^) is

(20) zt=z0+−z∗0+exp⁡−d2t+z∗t,t∈nτ,n+1τ,

we have lim_*t*→*∞*_ *z*(*t*) ≤ *μ*_2_/(1 − exp(−*d*_2_ *τ*)).

Furthermore, from (19), we have

(21) zmax=maxt∈R⁡zt=μ21−exp⁡−d2τ,zmin=mint∈R⁡zt=μ2exp⁡−d2τ1−exp⁡−d2τ.

The dose-delivery schedule of chemotherapy drugs can determinate their efficacy in killing cancer cells and degree of toxicity to the patients [23]. Besides, conventional chemotherapy drugs often have a therapeutic window which is defined as a range of a drug's serum concentration at which a desired effect occurs, below which there is little effect, and above which toxicity occurs [24]. Hence, we denote the least effective concentration (LEC) as *c*_min_ and the maximum tolerated concentration (MTC) as *c*_max_, respectively. Thus, we have *c*_min_ ≤ *z*_min_ < *z*_max_ ≤ *c*_max_. Without loss of generality, we suppose *c*_min_ = *z*_min_ and *c*_max_ = *z*_max_. Hence, we can get

(22) cmax=μ21−exp⁡−d2τ,cmin=μ2exp⁡−d2τ1−exp⁡−d2τ,

which is equivalent to

(23) μ2=cmax−cmin,τ=1d2ln⁡cmaxcmin.

Furthermore, we know that Adriamycin is a drug used in cancer chemotherapy and is commonly used in the treatment of a wide range of cancers, including hematological malignancies (blood cancers, like leukaemia and lymphoma), many types of carcinoma (solid tumors), and soft tissue sarcomas. In our research, we select Adriamycin as a chemotherapy drug and follow the dosage suggested by the manufactures of the Adriamycin drug [19]. The suggested procedure entails a single dose of 60–75 mg/m^2^ once every 21 days [25]. Further, we approximate mice to have surface area of 0.01 m^2^ and have body volume of 0.5357 L and use the dosing as 0.75 mg every 21 days. Supposing that Adriamycin is distributed uniformly in every tissues, we have

(24) μ2=0.750.5357=1.400 mg/L,τ=21  days.

Since Adriamycin has a half-life of about 48 hours, from the first equation of (18), we get

(25) zt=z0exp⁡−d2t⟹d2=1tln⁡z0zt

and then have

(26) $$\begin{array}{c} d_{2}=\frac{\ln2}{48/24}=\frac{\ln2}{2}=0.3466/day. \end{array}$$

Further, from (23), we can obtain

(27) cminμ2exp⁡d2τ−1=1.4exp⁡0.3466∗21−1=9.6688×10−4 mg/L,cmaxcminexp⁡d2τ=9.6688×10−4×exp⁡0.3466∗21=1.40096688 mg/L.

According to dosage suggested by the manufactures of the Adriamycin drug, we can get a single chemotherapeutic regimen:

(28) $$\begin{array}{c} TR_{C1}=\left(\;0,1.400\;mg/L,21\;\;days\;\right). \end{array}$$

**Figure 2:**
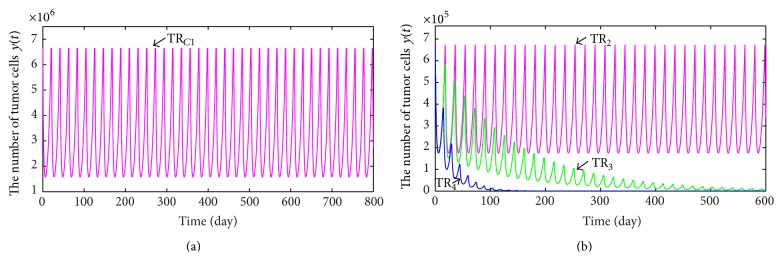
(a) presents the change of the number of tumor cells with time when treatment regimen is taken as TR_*C*1_; the variations of the number of tumor cells with time are shown in (b) when treatment regimes are TR_2_, TR_3_, and TR_4_.

Taking TR_*C*1_ as a chemotherapy regimen, the dynamic behavior of tumor cells is depicted in Figure 2(a) which indicates that the number of tumor cells exhibits periodic oscillation. In other words, single chemotherapeutic regimen TR_*C*1_ is not adequate to wipe out a tumor eventually. Since high concentration of chemotherapy drug can kill more tumor cells, we can try to elevate the average blood drug concentration of Adriamycin by increasing the least effective concentration of therapeutic window so that chemotherapy can obtain a better treatment result. In order to facilitate the following discussion, we denote new least effective concentration, new infusion dosage of Adriamycin, and new chemotherapy period by *c*_min_^*∗*^, *μ*_2_^*∗*^, and *τ* ^*∗*^, respectively. From (23), we have

(29) μ2∗=cmax−cmin∗,τ∗=1d2ln⁡cmaxcmin∗.

For different values of *c*_min_^*∗*^, from (29), we achieve different chemotherapy regimens which are listed in Table 2.

From Table 2, we clearly see that higher least effective concentration requires smaller infusion dosage of chemotherapy drug and shorter therapeutic period. By numeric calculations, we obtain a critical value of the least effective concentration *c*_min*c*_ = 0.002735 mg/L, which implies that if *c*_min_ > *c*_min*c*_, then the corresponding chemotherapy regimen is successful; otherwise chemotherapy regime is failing. Since 0.002735 mg/L and 0.004834 mg/L are more than *c*_min*c*_ and 0.002514 mg/L and 0.002611 mg/L are smaller than *c*_min*c*_, performing treatment regimens TR_3_ and TR_4_ can effectively control tumor growth, but executing therapeutic regimes TR_1_ and TR_2_ makes the number of tumor cells present periodical oscillation (see Figure 2(b)).

Supposing that the time of curing a cancer is still a year, we can obtain that the least effective concentration is not lower than *c*_min_ = 0.0080 mg/L. Hence, a single chemotherapy treatment regimen is given by (see Figure 2(b))

(30) $$\begin{array}{c} TR_{C2}=TR_{4}=\left(\;0,1.3930\;mg/L,\;\;14.90\;\;days\;\right). \end{array}$$

**Figure 3:**
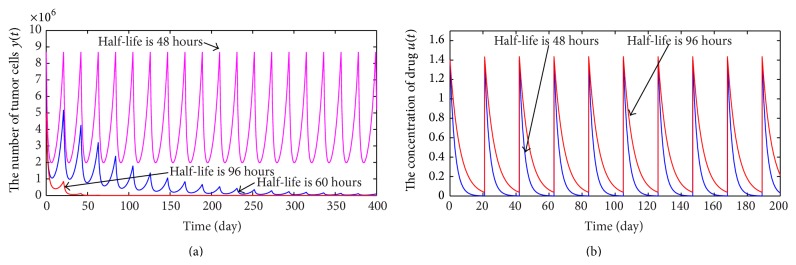
(a) presents the variations of the number of tumor cells with time when half-life of drugs is 48 h, 60 h, and 96 h, respectively; the changes of the drug concentrations with time are depicted in (b) when half times are 48 h and 96 h, respectively.

Further, we consider that the half-life of chemotherapy drug impacts on therapeutic result. Equation (25) indicates that extending the half-life of drug makes decay rate of chemotherapy drug reduce so that the average concentration of drug goes up. Thus, supposing that the half-life of Adriamycin is 48 h, 60 h, and 96 h, from (25), we obtain that the decay rates of it (*d*_2_) are 0.3466/day, 0.2773/day, and 0.1733/day, respectively. In addition, we still take TR_*C*1_ = (0,1.400 mg/L, 21  days) (i.e., recommended dosage suggested by the manufactures of the Adriamycin drug) as chemotherapy regimen. The variations of the number of tumor cells with time are depicted in Figure 3(a) when the half-lives of Adriamcin are 48 h, 60 h, and 96 h, respectively. The changes of the concentration of drugs with time are shown in Figure 3(b) when the half-lives of them are 48 h and 90 h, respectively. Figures 3(a) and 3(b) show that the half-life of drug is longer and the average concentration of drug is higher so that the effect of chemotherapy is better.

### 2.3. Mixed Immunotherapy with Chemotherapy

According to recommended dosage suggested by the manufactures of the Adriamycin drug, we know that the treatment regimen TR_*C*1_ is not sufficient to wipe out a tumor with size 5 × 10^6^ alone. In addition, increasing the dosage of Adriamycin may cause grievous damage to cancer patients. However, in contrast with chemotherapy, the immunotherapy can differentiate between normal and malignant cells and, thus, decrease the damage to normal cells or tissues due to chemotherapy. Hence, we consider mixed immunotherapy with chemotherapy and aim at designing a mixed treatment schedule which can prevent the tumor growth more effectively. In detail, we still take suggested dosage of the Adriamycin drug and try to seek out a critical dosage of CTL cells *μ*_1*Mc*1_, so that the tumor will die out eventually when *μ*_1_ > *μ*_1*Mc*1_. With the help of computer, we get *μ*_1*Mc*1_ = 136686 CTL cells. As a result, we achieve a mixed treatment regimen:

(31) $$\begin{array}{c} TR_{M1}=\left(\;136688\;CTL\;\;cells,1.4\;mg/L,21\;\;days\;\right). \end{array}$$

**Figure 4:**
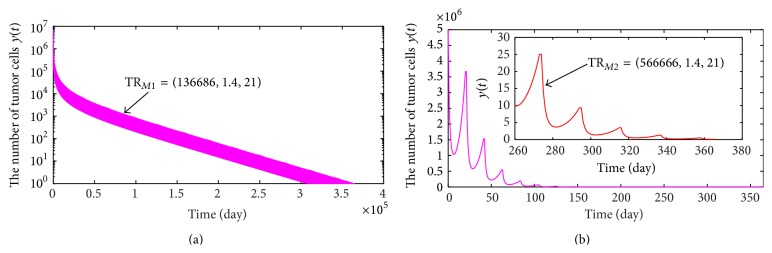
(a) shows the change of the number of tumor cells for treatment regimen TR_*M*1_; (b) presents the variation of the number of tumor cells of therapeutic regimen TR_*M*2_.

The dynamic behavior of tumor cells is shown in Figure 4(a) for therapeutic regimen TR_*M*1_. Figure 4(a) shows that although taking treatment regimen TR_*M*1_ can eliminate the tumor eventually, this process also takes too long time (up to 100 years). Obviously, TR_*M*1_ is not a good treatment means to eliminate a tumor with size 5 × 10^6^. In order to shorten the time of curing a cancer to a year, we need add infusion dosage of CTL cells. By numerical simulations, we achieve a new critical value *μ*_1*Mc*2_ = 566666 CTL cells. In other words, as long as infusion dosage of CTL cells is not less than *μ*_1*Mc*2_, then a tumor with size 5.00 × 10^6^ can be cured within a year (see Figure 4(b)). Thus, a new mixed treatment regimen is given by

(32) $$\begin{array}{c} TR_{M2}=\left(\;566666\;\;CTL\;\;cells,1.400\;mg/L,\;\;21\;\;days\;\right). \end{array}$$

Further, we also find that mixed immunotherapy and chemotherapy can make the number of tumor cells always in a lower level, which indicates that mixed treatment is evidently superior to single chemotherapy or single immunotherapy.

## 3. Considering the Cases of Drug Resistance

Although chemotherapies are effective treatment for metastatic tumors, the ability of cancer cells to become resistant to chemotherapy drugs remains a significant impediment to successful chemotherapy [26]. Drug resistance results from a variety of factors including individual variations in patients and somatic cell genetic differences in tumors, even those from the same tissue of origin. The most common reason for acquisition of resistance to a broad range of anticancer drugs is expression of one or more energy-dependent transporters that detect and eject anticancer drugs from cells, but other mechanisms of resistance including insensitivity to drug-induced apoptosis and induction of drug-detoxifying mechanisms probably play an important role in acquired anticancer drug resistance [27]. Hence, in this section, we will investigate the impact of drug resistance on therapeutic results and start a discussion about chemotherapy with a single drug at first.

### 3.1. Chemotherapy with a Single Drug

We introduce drug resistance into model (1) to explain the cause of chemotherapy failure with single drug. New model is given by

(33) dxdt=s+ρxy1+y2α+y1+y2−c1xy1+y2−d1x−α11−e−zx,t≠nτ,dy1dt=ry11−by1+y2−c2xy1−α3y1−α21−e−zy1,t≠nτ,dy2dt=α3y1+ry21−by1+y2−c2xy2,t≠nτ,dzdt=−d2z,t≠nτ,xt+=xt,t=nτ,yt+=yt,t=nτ,zt+=zt+μ2,t=nτ,

where *y*_1_ denotes the number of tumor cells which are sensitive to Adriamycin. *y*_2_ presents the number of tumor cells which are resistant to Adriamycin. *α*_3_ is the conversion rate of tumor cells from being sensitive to being resistant to Adriamycin. Usually *α*_3_ is very small since cancer cells mutate at a rate of about 1 in every 10^6^ cells; that is, *α*_3_ = 10^−6^ [28].

**Figure 5:**
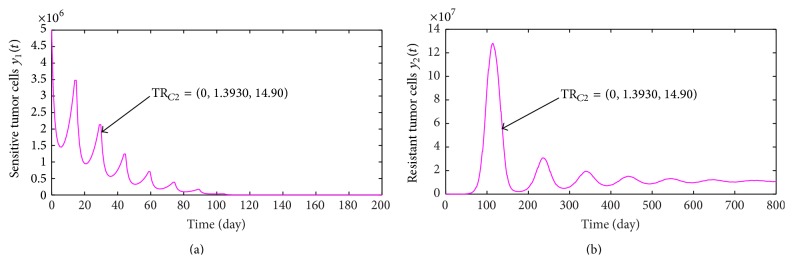
(a) and (b) present the variations of the number of sensitive tumor cells and resistant tumor cells with time for treatment regime TR_*C*2_, respectively.

As mentioned before, taking single chemotherapy regimen TR_*C*2_ can wipe out a tumor with size 5.0 × 10^6^ within a year without consideration of drug resistance. Now, we still take TR_*C*2_ as a treatment regimen. By numerical simulations, the variations of the numbers of sensitive tumor cells and resistant tumor cells are shown in Figures 5(a) and 5(b), respectively. From Figure 5(a), we know that the number of sensitive tumor cells to Adriamycin reaches 0 quickly. However, Figure 5(b) indicates that the number of resistant tumor cells to Adriamycin is stable at a fixed value (about 1.60 × 10^6^). All these findings show that the sensitive tumor cells will completely convert into resistant tumor cells as chemotherapy is executed, which implies that the drug resistance is the main cause of chemotherapy failure. In order to solve the problem of drug resistance, next, we will investigate the treatment effect of combination chemotherapy with two drugs.

### 3.2. Combination Chemotherapy with Two Drugs

As analyzed above, we know that the designs of cancer chemotherapeutic regimens have become increasingly sophisticated, and a single chemotherapy drug is very difficult to cure a tumor. The use of multiple therapeutic agents in combination has become the primary strategy to treat drug resistant cancers. This approach is called combination chemotherapy which provides a higher chance of destroying cancer cells. As a consequence, chemotherapy with two or more cytotoxic drugs that kill tumor cells by one or more mechanisms will be considered. To make the discussion easier, we assume that two drugs are administered to treat a tumor with drug resistance.

For convenience, we denote two chemotherapy drugs by *DA* and *DB* which are not toxic for the same normal organ. The concentrations of them are denoted by *z*_1_ and *z*_2_. *d*_2_ and *d*_3_ are the decay rates of them, respectively. The tumor cells population is divided into three subpopulations which are sensitive to drug *DA* and drug *DB*, sensitive to drug *DA* but resistant to drug *DB*, and sensitive to drug *DB* but resistant to drug *DA*, respectively. The numbers of them are denoted by *y*_1_, *y*_2_, and *y*_3_. Model (33) can be modified in the following way to account for combination chemotherapy:

(34) dxdt=s+ρxy1+y2+y3α+y1+y2+y3−c1xy1+y2+y3−d1x−α11−e−z1x−α51−e−z2x,t≠nτ,dy1dt=ry11−by1+y2+y3−c2xy1−α3+α4y1−α21−e−z1y1−α61−e−z2y1,t≠nτ,dy2dt=α3y1+ry21−by1+y2+y3−c2xy2−α61−e−z2y2,t≠nτ,dy3dt=α4y1+ry31−by1+y2+y3−c2xy3−α21−e−z1y3,t≠nτ,dz1dt=−d2z1,t≠nτ,dz2dt=−d3z2,t≠nτ,xt+=xt,t=nτ,y1t+=y1t,y2t+=y2t,y3t+=y3t,t=nτ,z1t+=z1t+μ2,z2t+=z2t+μ3,t=nτ,

where *α*_1_ and *α*_5_ are the fractional immune cells killed by drugs *DA* and *DB*. *α*_2_ and *α*_6_ are the fractional tumor cells killed by drugs *DA* and *DB*. *α*_3_ and *α*_4_ are the conversion rates of tumor cells from being sensitive to being resistant to drugs *DA* and *DB*. As mentioned above, *μ*_2_ and *μ*_3_ represent increments of the blood drug concentrations caused by combination chemotherapy every time. Analogously, we use TR_*Ri*_ = (*μ*_2*i*_, *μ*_3*i*_, *τ*_*i*_) to denote the *i*th combination chemotherapy regimen.

To facilitate discussion, we suppose that dynamical features and therapeutic effects of drugs *DA* and *DB* are the same as Adriamycin chemotherapy drug. Hence, we have *α*_1_ = *α*_5_ = 3.40 × 10^−2^, *α*_2_ = *α*_6_ = 0.90 × 10^−1^, and *d*_2_ = *d*_3_ = 3.466 × 10^−1^. The conversion rates of tumor cells from being sensitive to being resistant *α*_3_ and *α*_4_ are still taken as the values *α*_3_ = *α*_4_ = 10^−6^. The meanings and estimated values of all remaining parameters are the same as model (1). Next, in order to investigate the impact of the difference of two-drug dosages on therapeutic results, we consider two different modes, one case is *μ*_2_ = *μ*_3_, and the other is *μ*_2_ ≠ *μ*_3_.

**Case 1 (*μ*_2_ = *μ*_3_).**

To make tumor cells die out completely within a year and the concentrations of drugs be in a therapeutic window simultaneously, by calculating numerically, we achieve a new critical value of the least effective concentration, *c*_min*c*_ = 0.002901. That is, the tumor cells will be eliminated within a year if the least effective concentration is not less than *c*_min*c*_. Hence, by (23), we obtain a combination chemotherapy regimen:

(35) $$\begin{array}{c} TR_{R0}=\left(\;1.3971\;mg/L,1.3971\;mg/L,16.9290\;\;days\;\right). \end{array}$$

Comparing the therapeutic regimen TR_*R*0_ with TR_*C*2_ (see (30)), it is not difficult to find that curing a tumor with drug resistance is required to administrate more dosage of chemotherapy drug, which indicates that drug resistance is one of the difficult questions of tumor treatment.

When shortening therapeutic period to 10 days and requiring to cure a tumor within a year, by calculating, then we get another combination chemotherapy regimen:

(36) $$\begin{array}{c} TR_{R1}=\left(\;0.8000\;mg/L,0.8000\;mg/L,10.0000\;\;days\;\right). \end{array}$$

**Figure 6:**
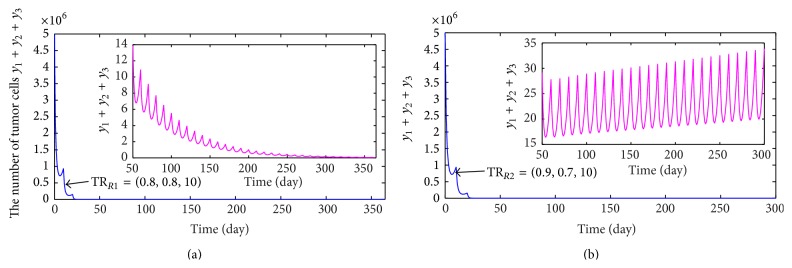
(a) and (b) describe the variances of the number of tumor cells when taking the combination chemotherapy regimens TR_*R*1_ and TR_*R*2_, respectively.

Executing treatment regimen TR_*R*1_, the variation of the number of tumor cells with time is shown in Figure 6(a), which shows that the number of tumor cells is always in a lower level and reaches 0 eventually. In the following, we will discuss another case.

**Case 2 (*μ*_2_ ≠ *μ*_3_).**

Here, we take delivering dosages of drugs *DA* and *DB* as 0.9000 mg/L and 0.7000 mg/L every 10 days, respectively. Thus, we obtain another combination treatment regimen:

(37) $$\begin{array}{c} TR_{R2}=\left(\;0.9000\;mg/L,0.7000\;mg/L,10.0000\;\;days\;\right). \end{array}$$

The dynamic behavior of tumor cells for treatment regimen TR_*R*2_ is exhibited in Figure 6(b). However, Figure 6(b) shows that the number of tumor cells is in a lower level at the early stage but later increases gradually. In other words, taking the therapeutic regimen TR_*R*2_ easily causes the tumor recurrence.

Cases 1 and 2 indicate that therapeutic effect of TR_*R*1_ is obviously superior to that of TR_*R*2_ although the total amount of delivering drug of TR_*R*1_ is the same as that of TR_*R*2_. Hence, a topic of how to determine the delivering dosage of every chemotherapy drug deserves deep exploration. The analysis result indicates that combination chemotherapy with multidrugs is very effective in controlling tumor growth, but larger dosage of chemotherapy drugs is destined to bring greater harm to normal tissues. As a result, in order to diminish the damage of chemotherapy to normal tissues, we will combine immunotherapy with combination chemotherapy.

### 3.3. Mixed Immunotherapy with Combination Chemotherapy

We substitute the seventh equation of model (34) by *x*(*t* ^+^) = *x*(*t*) + *μ*_1_ to analyze a new mixed immunotherapy with combination chemotherapy model. For convenience, we denote new therapeutic regimen as a quad TR_*MCi*_ = (*μ*_1*i*_, *μ*_2*i*_, *μ*_3*i*_, *τ*_*i*_), where the meanings of *μ*_*ji*_, *j* = 1,2, 3, and *τ*_*i*_ are the same as above. Since two chemotherapy drugs *DA* and *DB* are all toxic to normal cells, we assume delivering dosage is 80% of recommend dosage of Adriamycin. Thus, we have *μ*_2_ = *μ*_3_ = 1.4000 × 0.8 = 1.1200 mg/L. Hence, we obtain a combination chemotherapy regimen:

(38) $$\begin{array}{c} TR_{MC1}=\left(\;0,1.1200\;mg/L,1.1200\;mg/L,21\;\;days\;\right). \end{array}$$

**Figure 7:**
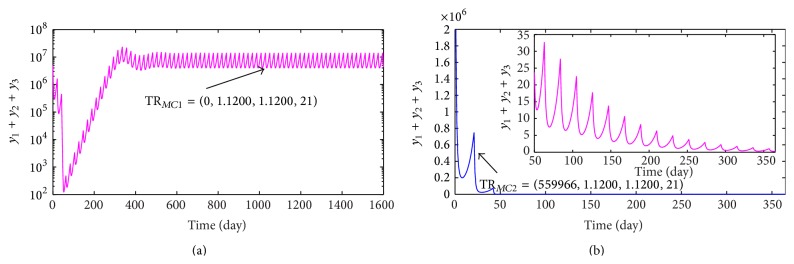
(a) and (b) present the variances of the number of tumor cells when taking the combination chemotherapy regimens TR_*MC*1_ and TR_*MC*2_, respectively.

When treatment regimen TR_*MC*1_ is performed, the variation of the number of tumor cells with time is exhibited in Figure 7(a) which shows that the number of tumor cells presents periodical oscillation. In other words, executing TR_*MC*1_ can not cure a tumor successfully. Based on this, we introduce immunotherapy into treatment regimen TR_*MC*1_. By numerical calculation, we achieve a threshold value *μ*_1*c*3_ = 559966 CTL cells. In detail, tumor will be eliminated within a year if infusion dosage of CTL cells is not less than *μ*_1*c*3_. Thus, a mixed immunotherapy with combination chemotherapy regimen is given by

(39) $$\begin{array}{c} \begin{array}{c} TR_{MC2}=\left(559966\;\;CTL\;\;cells,1.1200\;mg/L,1.1200\;mg/L,21\;\;days\right). \end{array} \end{array}$$

The dynamic behavior of tumor cells is shown in Figure 7(b) when the mixed treatment regimen TR_*MC*2_ is carried out. From Figure 7(b), we can obviously see that a tumor with size 5.00 × 10^6^ can be eliminated very quickly.

## 4. Conclusion

In this paper, we investigate the therapeutic effects of single immunotherapy, single chemotherapy, and mixed treatment and provide corresponding therapeutic regimens. For single immunotherapy, we derive a condition under which tumor cells will be eliminated ultimately, but this process may take too long time. Thus, we suppose that the time of curing a tumor is a year and then achieve a single immunotherapy regimen TR_*I*1_. For single chemotherapy, we select Adriamycin as chemotherapy drug. By exploring the impacts of the least effective concentration and the drug half-life on therapeutic results, we draw a conclusion that increasing the least effective concentration and extending the half-life of the drug can make average drug concentration maintain a higher level so that the effect of chemotherapy is better. Using numerical calculations, we obtain a threshold value *c*_min*c*_; that is, single chemotherapy can eliminate a tumor within a year if the least effective concentration is not lower than *c*_min*c*_. As a result, we obtain an ideal single chemotherapeutic regimen TR_*C*2_. Further, in order to decrease the damage of chemotherapy drug to normal tissues, we consider mixed immunotherapy with chemotherapy. Taking recommended dosage of Adriamycin and supposing the time of curing a tumor with size 5.0 × 10^6^ as a year, by numerical calculations, we achieve a mixed immunotherapy with immunotherapy regimen TR_*M*2_. In addition, we find that mixed treatment can make the number of tumor cells be always in a lower level.

Since most tumors are resistant to chemotherapy drugs, we consider influence of drug resistance on therapeutic results and improve a new mathematical model. By analyzing the target model, we explain the cause of chemotherapeutic failure. Further, we consider the effect of combination chemotherapy with two drugs and form a combination chemotherapy regimen TR_*R*0_ which can get rid of a tumor within a year. Finally, in order to cure a tumor more effectively, we consider mixed immunotherapy with combination chemotherapy to treat a tumor and establish an ideal mixed treatment regimen TR_*MC*2_ which can make tumor cells be always in a lower level and be also wiped out completely in a year.

In a word, combination chemotherapy is very effective in controlling tumor growth, and further mixed immunotherapy with combination chemotherapy can obtain a better treatment effect. But, with tumor cells becoming resistant to many structurally and mechanistically unrelated drugs, the efficacy of chemotherapy of tumor often becomes severely limited. Hence, the problem of how to combine reasonably those treatment modes and design an optimal mixed therapeutic regimen deserves deep research.

## Figures and Tables

**Table 1:** Estimated parameter values.

Parameter	Description	Estimated value	Source
*s*	Normal rate of flow of immune cells into the tumor site	1.300 × 10^4^	Kuznetsov et al. 1994 [16]
*ρ*	Maximum immune cells recruitment rate	1.254 × 10^−1^	Kuznetsov et al. 1994 [16]
*α*	Steepness coefficient of immune cell recruitment	2.020 × 10^7^	Kuznetsov et al. 1994 [16]
*c*_1_	Immune cells death rate due to interaction with tumor cells	3.420 × 10^−10^	Kuznetsov et al. 1994 [16]
*d*_1_	Nature death rate of immune cells	4.120 × 10^−2^	Kuznetsov et al. 1994 [16]
*α*_1_	Fractional immune cells kill by chemotherapy	3.400 × 10^−2^	de Pillis et al. 2006 [1]
*r*	Tumor cells growth rate	1.800 × 10^−1^	Kuznetsov et al. 1994 [16]
*b*	1/*b*_1_ is tumor cells carrying capacity	2.000 × 10^−9^	Kuznetsov et al. 1994 [16]
*c*_2_	Fractional tumor cells kill by immune cells	1.100 × 10^−7^	Kuznetsov et al. 1994 [16]
*α*_2_	Fractional tumor cells kill by chemotherapy	9.000 × 10^−1^	de Pillis et al. 2006 [1]
*d*_2_	Rate of chemotherapy drug decay	3.466 × 10^−1^	Estimated

**Table 2:** Different values of *c*_min_^*∗*^ and corresponding chemotherapy regimes.

*c*_min_^*∗*^	*μ*_2_^*∗*^	*τ* ^*∗*^	Chemotherapy regimens
0.002514 mg/L	1.3985 mg/L	18.24 days	TR_1_ = (0, 1.3985 mg/L, 18.24 days)
0.002611 mg/L	1.3984 mg/L	18.13 days	TR_2_ = (0, 1.3984 mg/L, 18.13 days)
0.002735 mg/L	1.3982 mg/L	18.00 days	TR_3_ = (0, 1.3982 mg/L, 18.00 days)
0.008000 mg/L	1.3930 mg/L	14.90 days	TR_4_ = (0, 1.3930 mg/L, 14.90 days)
